# Supplementary material for: Unveiling the Bioactive Potential of the Invasive Jellyfish Phyllorhiza punctata Through Integrative Transcriptomic and Proteomic Analyses
Source: Biomolecules. 2025 Aug 4;15(8):1121. doi: 10.3390/biom15081121 (PMC12383608; doi:10.3390/biom15081121)
Supplement: Supplementary file 1 [file biomolecules-15-01121-s001.zip › Suplementary Material/FileS3/PEPFOLD4/XP_065070517_12324/clusters.html]

 PEP-FOLD clusters


Download archive of all models  here   

|  |  |  |
| --- | --- | --- |
| # | file | sOPEP |
| # cluster 1 - Size: 3 |  |  |
|  | XP\_065070517\_12324-model1 | -10.0756 |
|  | XP\_065070517\_12324-model1.1 | -10.0756 |
|  | XP\_065070517\_12324-model1.2 | -10.0756 |
| # cluster 2 - Size: 22 |  |  |
|  | XP\_065070517\_12324-model2 | -6.14227 |
|  | XP\_065070517\_12324-model2.1 | -6.1254 |
|  | XP\_065070517\_12324-model2.2 | -6.1254 |
|  | XP\_065070517\_12324-model2.3 | -6.06383 |
|  | XP\_065070517\_12324-model2.4 | -6.06383 |
|  | XP\_065070517\_12324-model2.5 | -6.06383 |
|  | XP\_065070517\_12324-model2.6 | -6.06383 |
|  | XP\_065070517\_12324-model2.7 | -6.06383 |
|  | XP\_065070517\_12324-model2.8 | -6.06383 |
|  | XP\_065070517\_12324-model2.9 | -6.05186 |
|  | XP\_065070517\_12324-model2.10 | -5.93296 |
|  | XP\_065070517\_12324-model2.11 | -5.90958 |
|  | XP\_065070517\_12324-model2.12 | -5.89739 |
|  | XP\_065070517\_12324-model2.13 | -5.72713 |
|  | XP\_065070517\_12324-model2.14 | -5.69346 |
|  | XP\_065070517\_12324-model2.15 | -5.65516 |
|  | XP\_065070517\_12324-model2.16 | -5.49905 |
|  | XP\_065070517\_12324-model2.17 | -5.4212 |
|  | XP\_065070517\_12324-model2.18 | -5.39916 |
|  | XP\_065070517\_12324-model2.19 | -5.33357 |
|  | XP\_065070517\_12324-model2.20 | -5.12226 |
|  | XP\_065070517\_12324-model2.21 | -4.58268 |
| # cluster 3 - Size: 26 |  |  |
|  | XP\_065070517\_12324-model3 | -4.05997 |
|  | XP\_065070517\_12324-model3.1 | -4.05997 |
|  | XP\_065070517\_12324-model3.2 | -4.0516 |
|  | XP\_065070517\_12324-model3.3 | -4.05061 |
|  | XP\_065070517\_12324-model3.4 | -4.05061 |
|  | XP\_065070517\_12324-model3.5 | -4.03727 |
|  | XP\_065070517\_12324-model3.6 | -4.02305 |
|  | XP\_065070517\_12324-model3.7 | -4.02305 |
|  | XP\_065070517\_12324-model3.8 | -4.02305 |
|  | XP\_065070517\_12324-model3.9 | -4.00576 |
|  | XP\_065070517\_12324-model3.10 | -4.00303 |
|  | XP\_065070517\_12324-model3.11 | -3.98567 |
|  | XP\_065070517\_12324-model3.12 | -3.96666 |
|  | XP\_065070517\_12324-model3.13 | -3.90488 |
|  | XP\_065070517\_12324-model3.14 | -3.8986 |
|  | XP\_065070517\_12324-model3.15 | -3.88917 |
|  | XP\_065070517\_12324-model3.16 | -3.86167 |
|  | XP\_065070517\_12324-model3.17 | -3.86134 |
|  | XP\_065070517\_12324-model3.18 | -3.84243 |
|  | XP\_065070517\_12324-model3.19 | -3.82785 |
|  | XP\_065070517\_12324-model3.20 | -3.79182 |
|  | XP\_065070517\_12324-model3.21 | -3.78482 |
|  | XP\_065070517\_12324-model3.22 | -3.71262 |
|  | XP\_065070517\_12324-model3.23 | -3.51064 |
|  | XP\_065070517\_12324-model3.24 | -3.50711 |
|  | XP\_065070517\_12324-model3.25 | -3.48333 |
| # cluster 4 - Size: 18 |  |  |
|  | XP\_065070517\_12324-model4 | -4.03098 |
|  | XP\_065070517\_12324-model4.1 | -4.01212 |
|  | XP\_065070517\_12324-model4.2 | -4.00086 |
|  | XP\_065070517\_12324-model4.3 | -4.00086 |
|  | XP\_065070517\_12324-model4.4 | -3.96725 |
|  | XP\_065070517\_12324-model4.5 | -3.93187 |
|  | XP\_065070517\_12324-model4.6 | -3.93187 |
|  | XP\_065070517\_12324-model4.7 | -3.87927 |
|  | XP\_065070517\_12324-model4.8 | -3.8623 |
|  | XP\_065070517\_12324-model4.9 | -3.8354 |
|  | XP\_065070517\_12324-model4.10 | -3.8354 |
|  | XP\_065070517\_12324-model4.11 | -3.82196 |
|  | XP\_065070517\_12324-model4.12 | -3.81012 |
|  | XP\_065070517\_12324-model4.13 | -3.78931 |
|  | XP\_065070517\_12324-model4.14 | -3.64484 |
|  | XP\_065070517\_12324-model4.15 | -3.63467 |
|  | XP\_065070517\_12324-model4.16 | -3.57125 |
|  | XP\_065070517\_12324-model4.17 | -3.48102 |
| # cluster 5 - Size: 6 |  |  |
|  | XP\_065070517\_12324-model5 | -4.03069 |
|  | XP\_065070517\_12324-model5.1 | -4.02864 |
|  | XP\_065070517\_12324-model5.2 | -3.99543 |
|  | XP\_065070517\_12324-model5.3 | -3.9845 |
|  | XP\_065070517\_12324-model5.4 | -3.81974 |
|  | XP\_065070517\_12324-model5.5 | -3.59809 |
| # cluster 6 - Size: 8 |  |  |
|  | XP\_065070517\_12324-model6 | -4.01911 |
|  | XP\_065070517\_12324-model6.1 | -4.01911 |
|  | XP\_065070517\_12324-model6.2 | -4.0182 |
|  | XP\_065070517\_12324-model6.3 | -3.98445 |
|  | XP\_065070517\_12324-model6.4 | -3.97089 |
|  | XP\_065070517\_12324-model6.5 | -3.91734 |
|  | XP\_065070517\_12324-model6.6 | -3.77037 |
|  | XP\_065070517\_12324-model6.7 | -3.74708 |
| # cluster 7 - Size: 9 |  |  |
|  | XP\_065070517\_12324-model7 | -4.00574 |
|  | XP\_065070517\_12324-model7.1 | -4.00574 |
|  | XP\_065070517\_12324-model7.2 | -4.00203 |
|  | XP\_065070517\_12324-model7.3 | -3.99141 |
|  | XP\_065070517\_12324-model7.4 | -3.95171 |
|  | XP\_065070517\_12324-model7.5 | -3.94173 |
|  | XP\_065070517\_12324-model7.6 | -3.84986 |
|  | XP\_065070517\_12324-model7.7 | -3.55124 |
|  | XP\_065070517\_12324-model7.8 | -3.50821 |
| # cluster 8 - Size: 7 |  |  |
|  | XP\_065070517\_12324-model8 | -3.98718 |
|  | XP\_065070517\_12324-model8.1 | -3.74276 |
|  | XP\_065070517\_12324-model8.2 | -3.60199 |
|  | XP\_065070517\_12324-model8.3 | -3.59077 |
|  | XP\_065070517\_12324-model8.4 | -3.58175 |
|  | XP\_065070517\_12324-model8.5 | -3.53664 |
|  | XP\_065070517\_12324-model8.6 | -3.51922 |
| # cluster 9 - Size: 1 |  |  |
|  | XP\_065070517\_12324-model9 | -3.97956 |
|  |  |  |
